# An approach to the improvement of dissolution rate of inclusion complex of vat red 13 with β-cyclodextrin

**DOI:** 10.1038/s41598-025-92346-5

**Published:** 2025-03-18

**Authors:** Osama A. Hakeim, Fatma Abdelghaffar, Hamada M. Mashaly, Safia A. Mahmoud

**Affiliations:** National Research Centre, Textile Research and Technology Institute, Dokki, Cairo, Egypt

**Keywords:** Vat dyes, β-Cyclodextrin, Inclusion complex, Encapsulation, Reducing agent, Environmental sciences, Chemistry, Materials science

## Abstract

The dissolution rate of hydrophobic dyes like Vat Red 13 poses a significant challenge in Vat dyeing processes due to their poor solubility in aqueous media. This study explores the formation and characterization of an inclusion complex between Vat Red 13 (VR13) and β-cyclodextrin (β-CD) to enhance its dissolution rate. The solid inclusion complex is prepared using two distinct techniques: physical mixture, and encapsulation by adopting a molar ratio 1:1. The enhancement of inclusion complex solubility in the liquid state was examined using the UV-Vis absorption spectrum values. The solid inclusion complex was characterized using transmission electron microscope, Fourier transform infrared spectroscopy, dynamic light scattering, X-ray diffraction, and contact angle analysis. Results demonstrated a significant increase of absorption as well as obvious solubility of β-CD/VR13 compared to conventional VR13. The enhanced dissolution rate can be attributed to the encapsulation of VR13 into the hydrophobic cavity of β-cyclodextrin, which facilitates better interaction with the solvent. Moreover, the disruption of its crystalline structure, which led to the generation of more amorphous regions. The β-CD/VR13 inclusion complex minimized the amount of reducing agent needed by approximately 75% and sped up the vat dyeing process on viscose fabric. This approach offers a promising solution for improving the dyeing efficiency of Vat dyes in textile applications.

## Introduction

Cellulose is the most abundant and renewable biopolymer. Cellulose-based fibres are divided into natural fibres (flax, jute, Kapok^1^, and cotton), and regenerated fibres (viscose and lyocell)^2–5^. Viscose is a regenerated fibre derived from natural cellulosic sources, with a chemical structure similar to cotton^6–8^.

Vat dyes, an important dyes class, are used to colour cellulosic fabrics due to their high production quality, especially when excellent fastness standards such as washing, light, and chlorine bleaching are required^9–11^. Furthermore, from a marketing perspective, vat dyes have dominated the dyestuff market for cellulosic fibres coloration. Whereas, since 1992, the global consumption of vat dyes, including indigo, has been over 33,000 metric tonnes, contributing about 24% of the cellulosic fibre dye industry in value terms^12^.

On the other hand, vat dyes, including Vat Red 13, are insoluble in water, requiring reducing agents, alkali, dispersing agents, and other auxiliaries, including sodium dithionite, to convert the dispersed oxidized vat dye into its so-called soluble leuco-form, resulting in exhibiting affinity to cellulose fibres. Due to the use of that quantity of reducing agents and alkali in the dyebath, high chemical concentrations in wastewater are released, which leads to a huge eco-system problem, economic implications, and operational challenges^13–15^.

Commonly, sodium dithionite (Na_2_S_2_O_4_) is one of the most regularly applied reducing agents for vat dyeing. Despite this, sodium dithionite cannot be regenerated; and in addition, it is harmful to both the environment and humans. Its use results in a host of concerns in the dyeing wastewater because sodium dithionite eventually oxidizes to many compounds: including sodium sulphate, sulphate ions, and thiosulphate ions, which are all ecologically harmful owing to their toxicity. These byproducts contribute to water contamination by increasing the chemical oxygen demand (COD) and toxicity levels in wastewater. Furthermore, the widespread usage of alkalis exacerbates the environmental impact^9,16–18^. Further, using significant amounts of reducing agents and alkalis raises production costs for textile makers. Also, inefficiency induced by poor solubility leads to longer processing times, increased energy consumption, and increased material waste as a result of insufficient dye fixation^10,13^. Poor solubility also affects dyeing uniformity and penetration into cellulose fibers, resulting in variable color performance and poor dye consumption efficiency. This necessitates additional procedures, such as extended reduction or several dying cycles, which increase resource consumption and operational complexity.

Consequently, several initiatives are being essayed to replace the environmentally damaging sodium dithionite with eco-friendlier alternatives. Investigations were concentrated on either replacing sodium dithionite with an organic reducing agent (i.e., α-hydroxyketones) with biodegradable oxidation products or using ultrasound to expedite the vatting operation and boost conversion. Indigo catalytic hydrogenation is another well-known technique^19^. However, because of the significant risk of explosion and fire, this procedure cannot be used directly in the dye house. Another method to increase the solubility of vat dyes is the use of cyclodextrins. Cyclodextrins (CDs) are cyclic oligosaccharides composed of D-glucopyranose units linked by α-D-1–4-glycosidic bonds^20,21^. They feature a cavity structure with a hydrophobic interior and a hydrophilic exterior. These CDs form water-soluble intermolecular complexes with lipophilic organic compounds by encapsulating these molecules within their hydrophobic cavity, creating what is known as a “guest-host” complex^22–24^. The hydrophilic surface of CDs, rich in hydroxyl groups, enhances interactions with dyes via hydrogen bonding and electrostatic interactions^25,26^.

The aim of the present investigation is to improve the aqueous solubility of poorly water-soluble Vat Red 13 (VR13) by forming an inclusion complex with β-cyclodextrin (β-CD). This focus could be ecologically and economically acceptable and reflects on the environmental concern which inquires to reduce the use of environmentally unfavorable reducing agents in vat dyeing. β-CD/VR 13 inclusion complexes prepared by two different methods like kneading method and encapsulation^27,28^ method were characterized by Fourier Transform Infrared Spectroscopy, Transmission electron microscopy, Contact angle, and Particle size distribution analysis. The improvement of solubility of β-CD/VR 13 complexes compared to VR 13 was indicated from the absorption spectral values of UV-Vis measurements. The β-CD/VR 13 solid state has been applied in dyeing of viscose to evaluate the effect of β-CD on the reducing agent account and the difference of coloration performance of β-CD/VR13 and conventionally insoluble vat dye.

## Experimental

### Materials

**Scheme 1:**
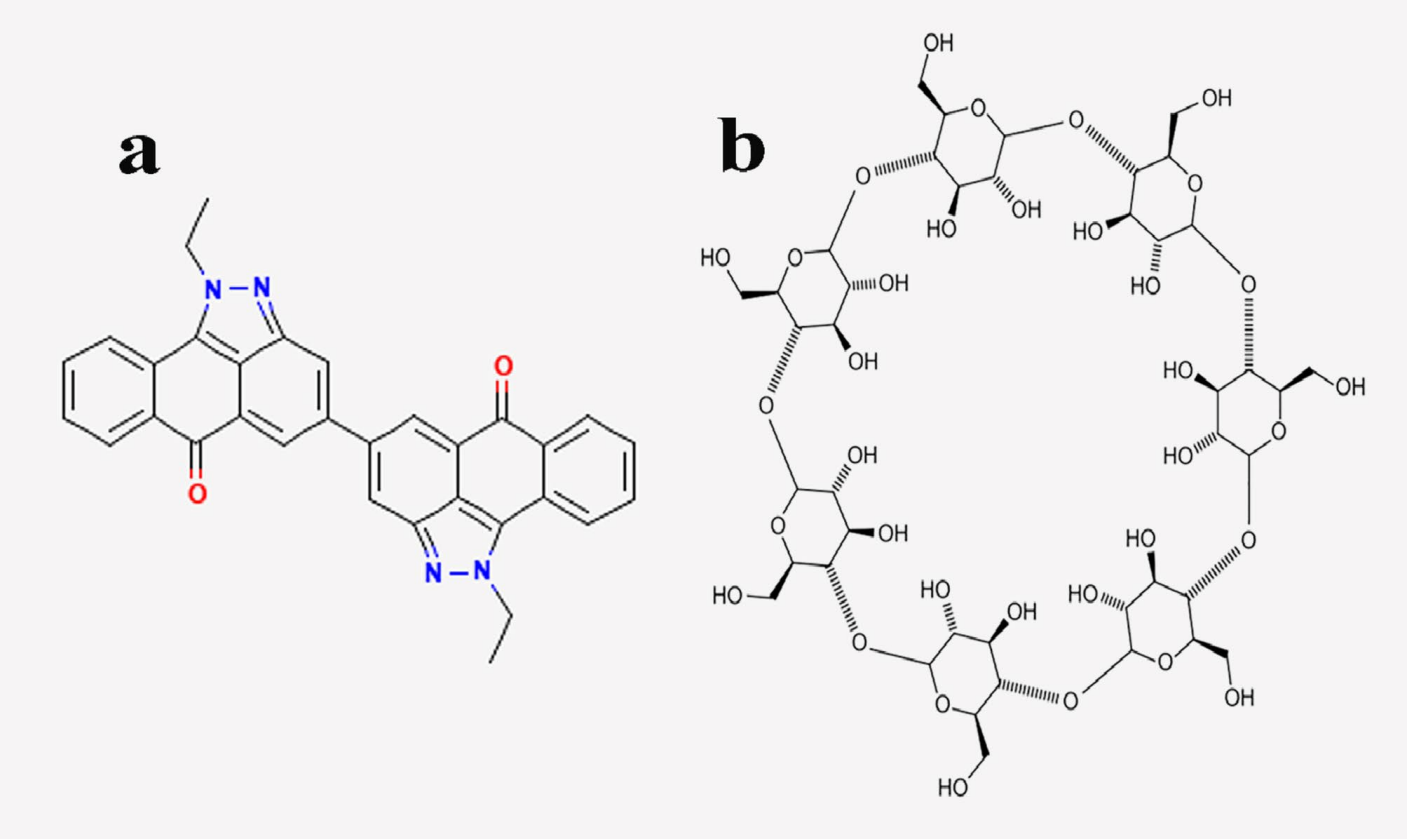
Structure representation of (**a**) chemical structure of Vat Red 13 and (**b**) chemical structure of β-CD.

100% viscose fabric were received from Misr spinning and weaving, Mahalla El-Kubra, Egypt. Vat Red 13 (Cibanone Red 6B, Molecular Formula: C_32_H_22_N_4_O_2_, supplied from Ciba Geigy) (Scheme 1a). Sodium hydrosulfite (a reducing agent) and sodium hydroxide were supplied from Sigma Aldrich Co. Chloroform was purchased from S D Fine-Chem limited Co. β-Cyclodextrin was obtained from ACROS ORGANICS (Scheme 1b). All other chemicals and reagents used were of the laboratory grade.

## Preparation of solid-inclusion complexes of β-CD/ VR 13 system

The solid inclusion complexes of VR 13 with β-CD were prepared using kneading and encapsulation methods by adopting the molar ratio of 1:1. In kneading method, β-CD/VR 13 complex was prepared by manual grinding of molar ratio of vat dye and β-CD in a mortar for about 30 min., additionally uses small volume of methanol/ water solution (1:1, v/v) to form a homogeneous paste of VR 13 and β-CD. The homogeneous paste was subjected to kneading for 30 min and then dried in an air oven at 70◦C for about 2 h. Finally, the dried β-CD/VR 13 inclusion complex was ground into fine powder. Liquid phase method has been carried out for encapsulation of VR 13 into β-CD. Molar ratio of β-CDX was dissolved in 160 ml of water; the same molar ratio of VR13 was added to the above solution under stirring. The mixture was dispersed at 10,000 rpm for 20 min with an Ultra-Turrax IKA T18 Basic instrument (IKA Instruments, Ltd., Staufen, Germany). Chloroform (80 ml) was mixed with the mixture at the rate of 5 ml/min. The resulting dispersion was centrifuged to produce slurry. Finally, the slurry was filtered, washed three times with distilled water; it was then dried at 60 °C to obtain β-CD-encapsulated Vat dye powder^24^.

## Vat dyeing

The vat dyeing procedure of viscose fabric with VR 13 was performed at 2% o.w.f. depth of shade in the presence of 5–20 g/l sodium hydrosulfite, 5–20 g/l sodium hydroxide at a liquor ratio 40:1the dye bath. The vat dyeing was then performed by raising the temperature of the dye bath from 40 °C to 60 °C, the dyed samples were then rinsed in distilled water for 5 min followed by dye oxidation using 2 g/l hydrogen peroxide, at a liquor ratio 20:1for 15–20 min. The dyed, oxidized fabrics were thoroughly rinsed with water.

## Color strength

The color strength results of the dyed samples were evaluated by Hunter lab Ultra Scan PRO and expressed as K/S.

## Fastness properties

The color fastness to crocking was determined according to AATCC 8–1993. Washing fastness tests were conducted according to the specified method, BS1006:C02. Test 2 with a soap solution (5 g/L, liquor ratio = 50:1) for 45 min at 48–50 °C. The light fastness test was carried out according to the standard methods for determination of color fastness of textiles using carbon-arc lamp^29,30^.

### Characterization

#### Transmission electron microscopy (TEM)

Morphology of β-CD/VR13 inclusion complex by kneading or encapsulation method and conventional VR13 was characterized by transmission electron microscope (TEM FEI MORGAGNI 268(D), Netherlands).

## Scanning electron microscopy (SEM)

The scanning electron microscopy (SEM) images were analyzed by JEOL (JAX-480 A).

### The size distribution

The size distribution was determined by Zetasizer Nano ZS (Malvern Instruments, Malvern, UK) operated by laser diffraction method.

### ^1^**H nuclear magnetic resonance analysis**.

^1^H NMR spectra were assessed with a JEOL device at 500 MHz, and DMSO-*d*_6_ as a solvent over TMS as standard.

### Fourier transform infrared spectroscopy (FTIR)

Fourie transform infrared spectra (FTIR spectra) of β-CD/VR13 (in KBr pellet) were recorded on a Spectrum BX FTIR spectrometer (Perkin-Elmer, USA). The FTIR absorption data were also used to see the changes in the structure of β-CD/VR13 solid like TCI (Total Crystallinity Index), CI-IR (crystallinity index) and HBI (Hydrogen Bond Intensity) parameters. The TCI parameter was measured by comparing the absorbance value at wavenumber 1373 and 2900 cm ^−1^ (A1373 / A2900) from the FTIR spectrum. The ratio of absorbance values at peak 1360.69 and 662.8 cm ^−1^ is the parameter for the CI-IR value (A1360.69 / A662.8). The HBI parameters were determined by the wavenumber ratio at the peak of 3400 and 1320 cm ^−1^(A3400 / 1320)^31,32^.

### The XRD

The X-ray diffraction (XRD) analyses were carried out by a Philips PW 3710 type diffractometer, Germany, equipped with a PW 3020 vertical goniometer and curved graphite diffracted beam monochromator. Average crystallite size (D(Å)) is estimated by the Debye-Scherrer Eqs^31,33^.

$$D=\frac{K\:{\it \lambda}}{\beta\:{\rm cos}\:\theta}$$

K(constant) = 0.89, ʎ= Wavelength for copper = 1.54 Ǻ, B (breadth) = Full-width half maximum (FWHM) (Radian), θ = Bragg angle. The degree of crystallinity (Xc) was estimated as described by Lorentzian function^33^. The crystallinity index.

(CI) was determined according to Segal method^34^.

### Contact angle

Contact angle measurements were performed using a compact video microscope manufactured by SDL-UK.

### UV-Vis spectroscopy

UV-Vis absorption values of β-CD/VR 13 with the increase of β-CD concentration from 0 to 0.06 M as well as the conventional VR 13, in aqueous medium at room temperature were measured using a Spectronic Unicam UV-300 UV/VIS Spectrometer, (Thermo Electron Corporation, made in England).

### Phase-solubility studies

Phase solubility studies were conducted in an aqueous medium at room temperature following the method described by Higuchi and Connors^35^. An excess amount of VR13 (50 mg) was introduced into 10 ml of distilled water, with β-CD concentrations gradually increasing from 0 to 0.06 M. The prepared suspensions were subjected to continuous shaking on a mechanical shaker for 24 h to reach equilibrium. Afterwards, the samples were filtered and appropriately diluted for spectrophotometric determination of VR13 concentration^36^. The stability constant (Ks) was derived from the phase solubility curve, assuming a 1:1 stoichiometric ratio, using Eq. (1):

1 $$\:Ks=Slope/\left({S}_{0}\right(1-Slope\left)\right)$$

where S_0_​ represents the solubility of VR13 in the absence of β-CD.

## Results and discussion

### UV-Vis absorption of β-CD/VR13

**Fig. 1:**
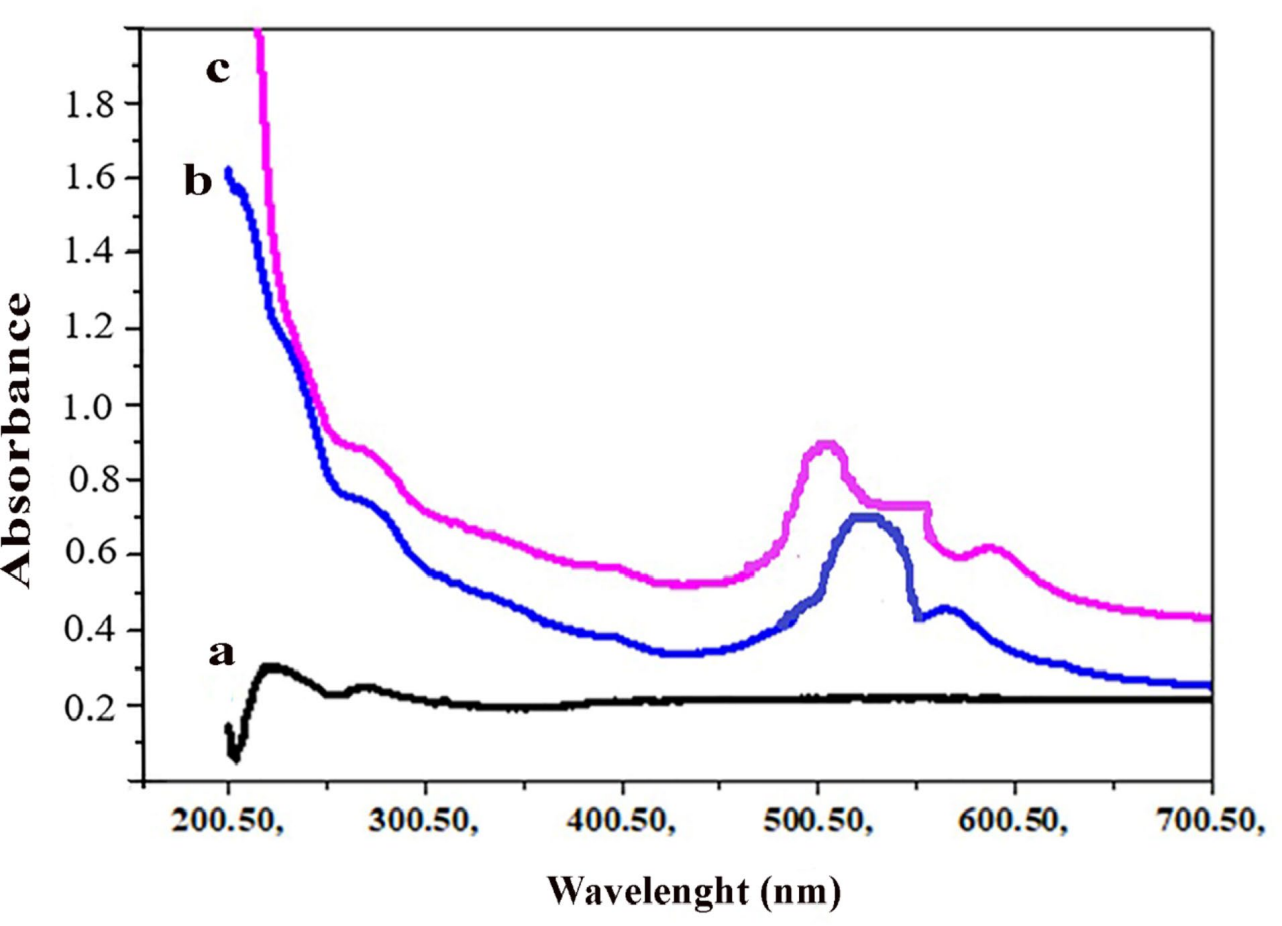
Absorption spectra of (**a**) β-CD, (**b**) VR13 and (**c**) β-CD/VR13 complex (kneading**)**.

**Fig. 2:**
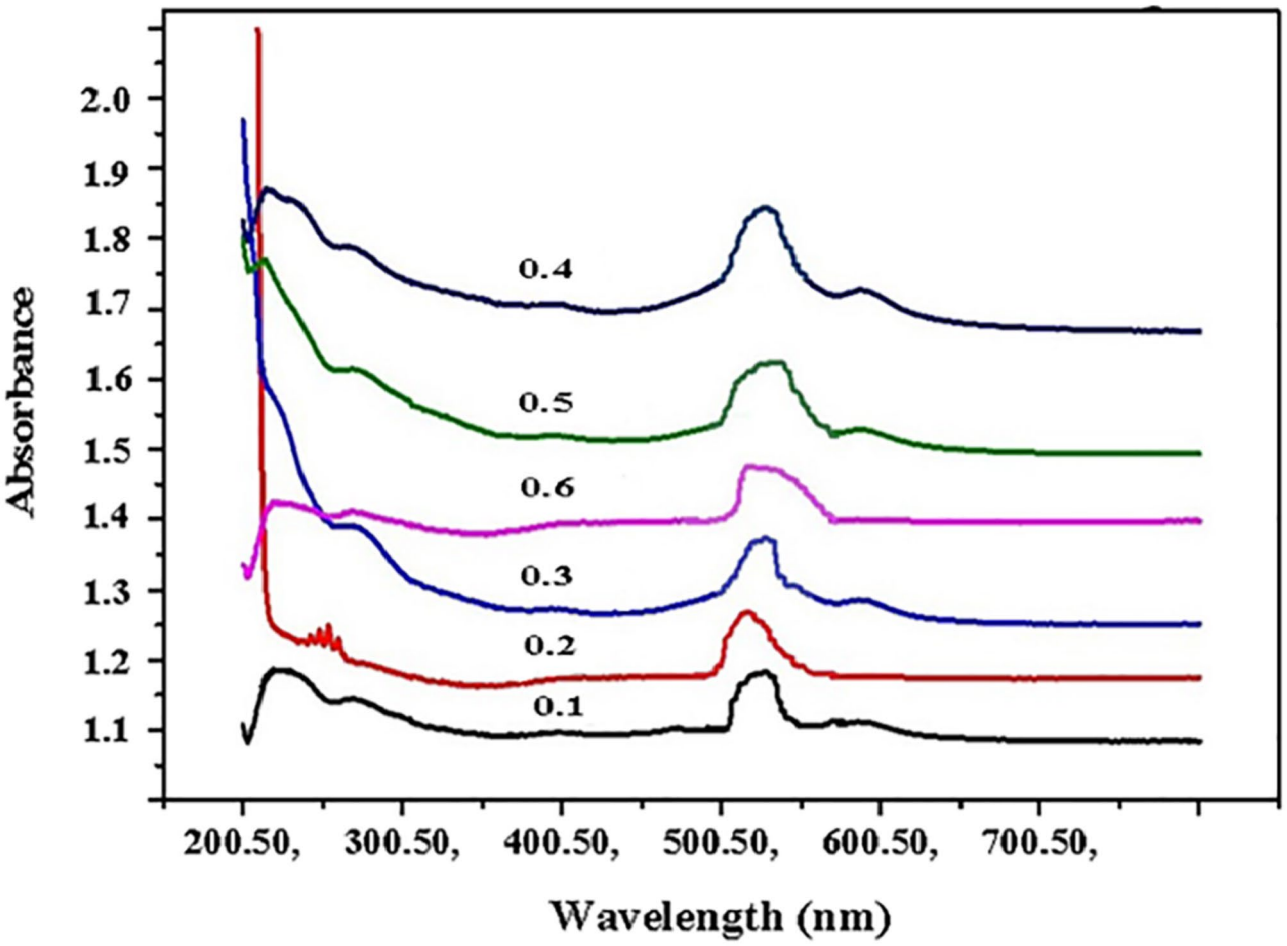
Absorption spectra of β-CD/VR13 complex with increasing concentration of β-CD using the kneading method.

**Scheme 2:**
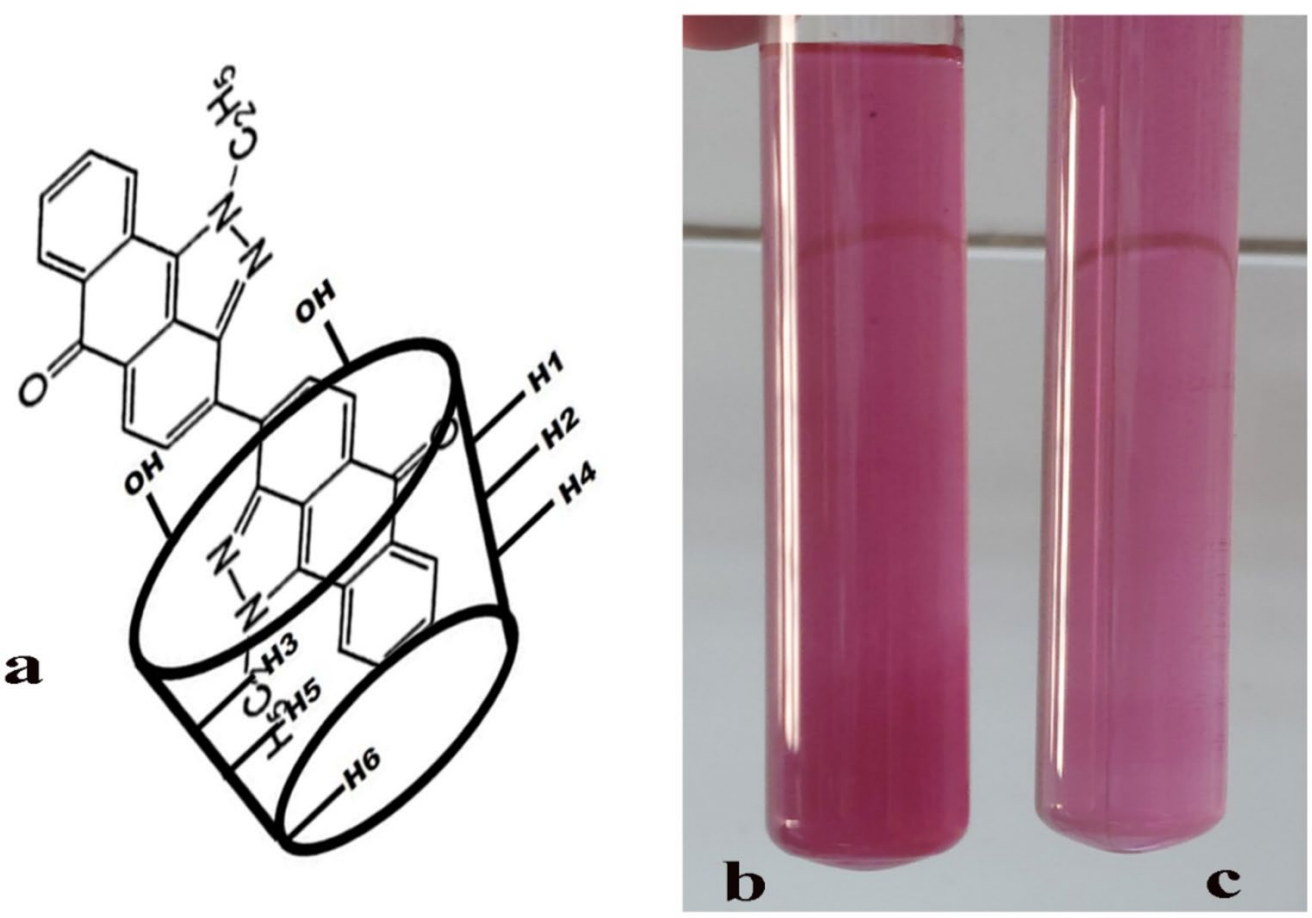
**(a)** Schematic representation of β-CD/VR13 inclusion complex, (**b**) the VR13 solution, (**c**) β-CD/VR13 solution.

The extent of interaction between the β-CD and VR13 was measured by UV-Vis absorbance values of VR 13 and β-CD/vat inclusion complex (Scheme 2) at different concentrations of β-CD. The results are shown in Table 1; Figs. 1 and 2. It has appeared that the absorption band of VR13 obtained a maximum peak at 520 nm, however, β-CD showed no absorbance due to the absence of any π-electrons or non-bonding electrons^37^. The spectral change upon complexation by kneading method with β-CD revealed slightly blue-shifted to a shorter wavelength near 500 nm, as described in Fig. 1c. The shift of absorption of VR13 upon addition of β-CD could be suggested the inclusion of VR13 into the host molecule, β-CD. It has also appeared that the absorbance of β-CD/VR13 exhibited hyperchromic effect, increasing the value of absorbance compared with original VR13. This may be attributed to the presence of hydrogen bonding and hydrophobic interactions^38^ between the β-CD and VR13. Table 1; Fig. 2 showed that the absorption value of β-CD/VR13 increased by increasing the concentration of β-CD. This may be due to the increase of molar absorptivity of VR13. These data could prove that the solubility of VR13 has been improved with the β-CD addition. In other words, the results indicated that the VR13 dissolution is increased with the function of β-CD concentrations. β-CD generates a hydrodynamic layer around VR13 molecules, increasing their local solubility during dissolution. In this regard, it was observed from Table 1that UV-Vis absorption measurements of β-CD/VR13 increased linearly as the concentration of β-CD in the range from 0.01 to 0.04 M, irrespective of the method of complexation, indicating a significant increase in solubility. For example, at a β-CD concentration of 0.04 M, absorbance increased from 0.935 for pure VR 13 to 1.799 for the kneading process and 1.599 for the encapsulation approach. This equates to a 92% (kneading) and 71% (encapsulation) increase in molar absorptivity, respectively. Whereas the concentration exceeds 0.04 M, the absorption of VR13 starts to decrease. This may be due to the formation of water insoluble material at higher concentrations of β-CD which in turn decreased the molar absorptivity^24^.

It should be mentioned that the enhancement of vat dye solubility which is relevant to the increase of molar absorptivity due to the amount of dissolved VR 13 increases quickly due to the β-CD molecules’ local solubilization impact, which creates a hydrodynamic layer around the VR13 molecules early in the dissolution process^39^. Another factor has been taken into account that the β-CD molecule possesses surfactant-like characteristics that can lower the interfacial tension between VR13 and the dissolving liquid. Generally speaking, the results of the molar absorptivity of the complex, suggesting a better inclusion by kneading method compared to encapsulation.

**Table 1:** Absorption maxima (nm) and its absorbance values of β-CD/VR13 complex at various concentrations of β-CD.

Sample	Conc. of β-CD (mmol)	λ_max_	A
**VR13**	0	500	0.7292
		520	0.935
**β-CD/VR13** **(kneading)**	0.01	500	1.1184
		520	1.194
	0.02	500	1.236
		520	1.289
	0.03	500	1.4256
		520	1.499
	0.04	500	1.768
		520	1.799
	0.05	500	1.608
		520	1.644
	0.06	500	1.566
		520	1.589
**β-CD/VR13** **(encapsulation)**	0.01	500	0.986
		520	1.111
	0.02	500	1.199
		520	1.291
	0.03	500	1.434
		520	1.499
	0.04	500	1.577
		520	1.599
	0.05	500	1.533
		520	1.557
	0.06	500	1.489
		520	1.499

From this figure (Fig. 1) we can see that absorbance value of VR13 + β-CD (kneading) higher than both of VR13 and β-CD. These explain the role of β-CD which plays in solubility of vat dye in water.

The clearer dissolution of Vat Red 13 after encapsulation into β-cyclodextrin could be visually supports the enhanced dissolution of Vat Red 13, which are directly attributed to the encapsulation process and the disrupted crystalline structure. Scheme 2 provides visual confirmation of the improved solubility of Vat Red 13 after encapsulation compared to conventional one. It demonstrates more uniform dispersion as well as a reduction in particle aggregation, as evidence of enhanced solubility.

### Phase solubility studies

**Fig. 3:**
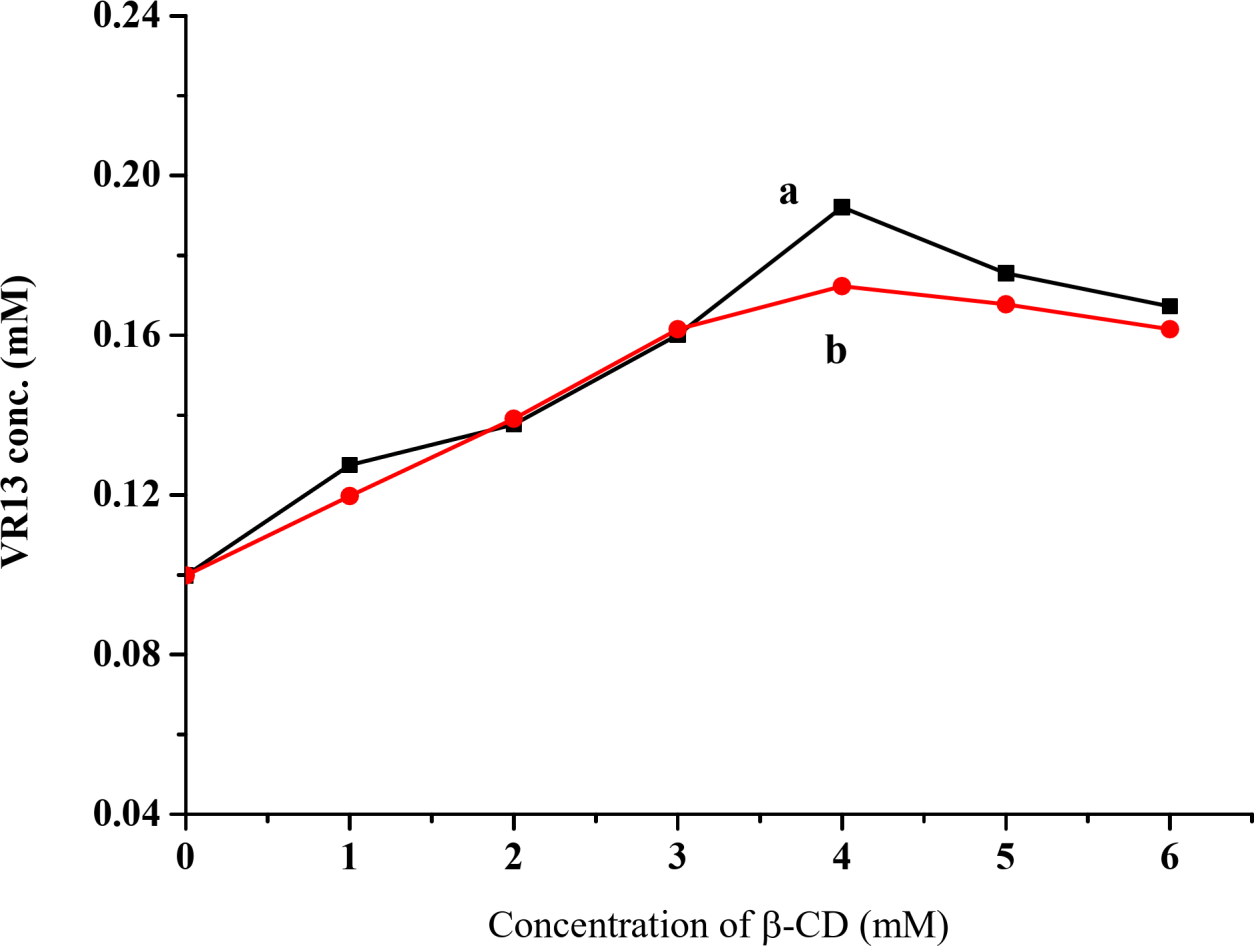
Phase solubility diagram of β-CD/VR13 in water for (**a**) kneading and (**b**) encapsulation method.

The phase solubility study is primarily used to determine the apparent stability constant of the inclusion complex. The phase solubility diagram of VR13 with β-CD is illustrated in Fig. 3; Table 1. The results indicate that the solubility of VR13 increases linearly with β-CD concentration in the range of 0 to 0.04 M. However, beyond 0.04 M, the solubility of VR13 begins to decline, possibly due to the formation of water-insoluble complexes at higher β-CD concentrations^40^. According to Higuchi and Connors, this solubility behavior is classified as a Bs-type system. The 1:1 molar ratio of the inclusion complex was determined from the initial linear portion of the solubility diagram, with a slope value of 0.9983 and 0.9282 for kneading and encapsulation methods, respectively. Based on Eq. (1), the apparent stability constant K1:1calculated were 147.9, 129.4 mol^−1^ for kneading and encapsulation methods. These results imply robust host-guest interactions, which contribute to increased dissolution.

### Characterization of β-CD/VR13 in solid state

#### FTIR

**Fig. 4:**
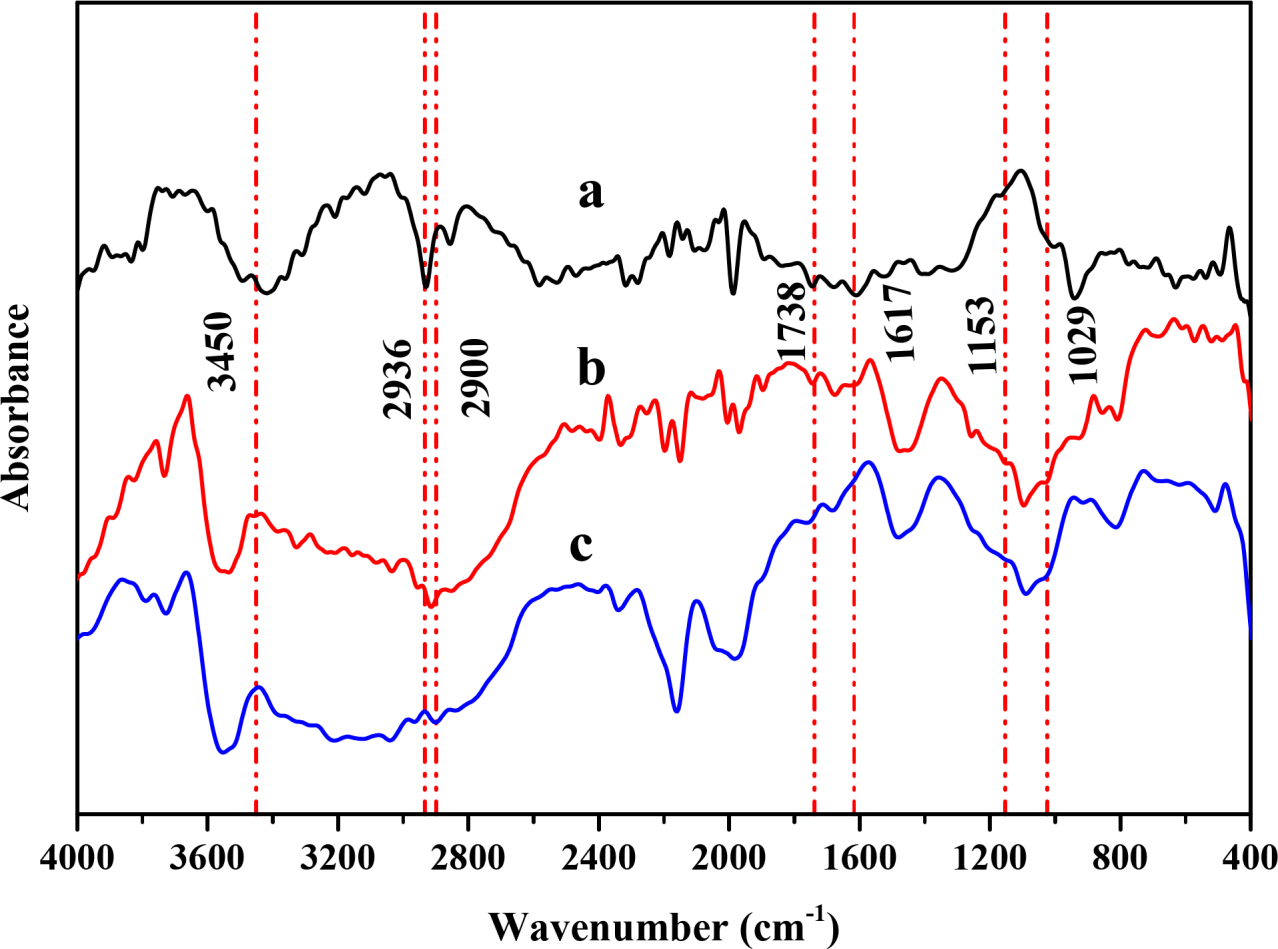
FTIR spectra of (**a**) VR13, (**b**) β-CD and (**c**) β-CD/VR13 complex by kneading method.

FT-IR spectroscopy is one method for approving the creation of solid-state complexes, this is because the peak’s intensity that corresponds to the part containing the guest molecules is shifted, decreased, or occasionally disappears. Figure 4 shows the characteristic peaks of VR13, β-CD, and its solid complexes prepared by the kneading method. The Blank (VR13) was characterized by the FT-IR, as shown in Fig. 4a, with the absorption bands at 1030 cm ^−1^ (C-N stretching vibration), 1617 cm ^−1^ (aromatic C = C stretching vibration), 1738 cm ^−1^ (C = O stretching vibration) 2900 cm ^−1^ (stretching vibration of -C-H).

Absorption bands at 3450 cm ^−1^ (stretching vibration of -OH), 2936 cm ^−1^ (stretching vibration of -C-H), and 1483 cm ^−1^ (stretching vibration of -OH deformation) were observed in the β-CD FT-IR spectra (Fig. 4b). Additionally, the H–O–H deformation bands of water found in β-CD were represented by a peak at 1650 cm ^−1^. The C–O–C vibration was absorbed at 1153 cm ^−1^, and the distinctive peak at 1029 cm ^−1^ suggested C-H, C-O stretching. The absorption vibrations of β-CD dominated the absorption band of the β-CD/VR13 complex upon inclusion. As a result, the FT-IR spectra of β-CD and the recorded FT-IR spectrum of the complex in Fig. 4c were quite similar. As expected, all the FTIR spectra of the inclusion complexes were identical with the β-CD spectrum. All the sharp peaks belonging to β-CD were observed. The characteristic peaks of VR13 disappeared (especially the absorption band at 1738 cm ^−1^ (C = O stretching vibration) and the absorption bands at 1030 cm ^−1^(C-N stretching vibration)). Based on these results, we inferred that the benzene rings of VR13 were contained within the β-CD cavity by van der Waals forces and hydrophobic interactions^41^. It is worthily to mention that most of the related β-CD peaks were shifted to higher or lower wave numbers in the spectrum of β-CD/VR13 complex. This may have confirmed the presence of β-CD in the inclusion complex. In other words, the shielding effect for the characteristic peaks of VR13 in the spectrum of β-CD/VR13 complex could give supporting evidence for the inclusion of VR13 into β-CD. Based on our experimental findings, the interaction can be attributed to the following mechanism: The hydrophobic cavity of β-CD creates an inclusion complex with VR13 using van der Waals forces and hydrophobic interactions, resulting in encapsulation. This encapsulation breaks VR13’s crystalline structure, changing it to a more amorphous state and increasing its solubility. These mechanisms show how the hydrophobic cavity of β-CD interacts with VR13 to enhance its dissolving rate and solubility.

The values of the TCI, CI-IR., and HBI substrates were then determined using the data from the FTIR spectra, as given in Table 2. β-CD/VR13 complex crystallinity was assessed using the values of TCI and CI-IR. Both TCI and CI-IR. values of VR13 decreased drastically after inclusion into β-CD. This is consistent with the crystallinity discovered using XRD patterns by Segal^34^or Lorentzian^33^. The HBI values of VR13, β-CD, and β-CD/VR13 were slightly different, but they increased after inclusion of VR13 into β-CD. The rise of HBI values after the inclusion of VR13 into β-CD is due to the production of a highly energetic amorphous state, which cannot preserve the VR13 structure. As a result, intra and intermolecular hydrogen bonds of β-CD become more exposed in the substrates.

### Contact angle

**Fig. 5:**
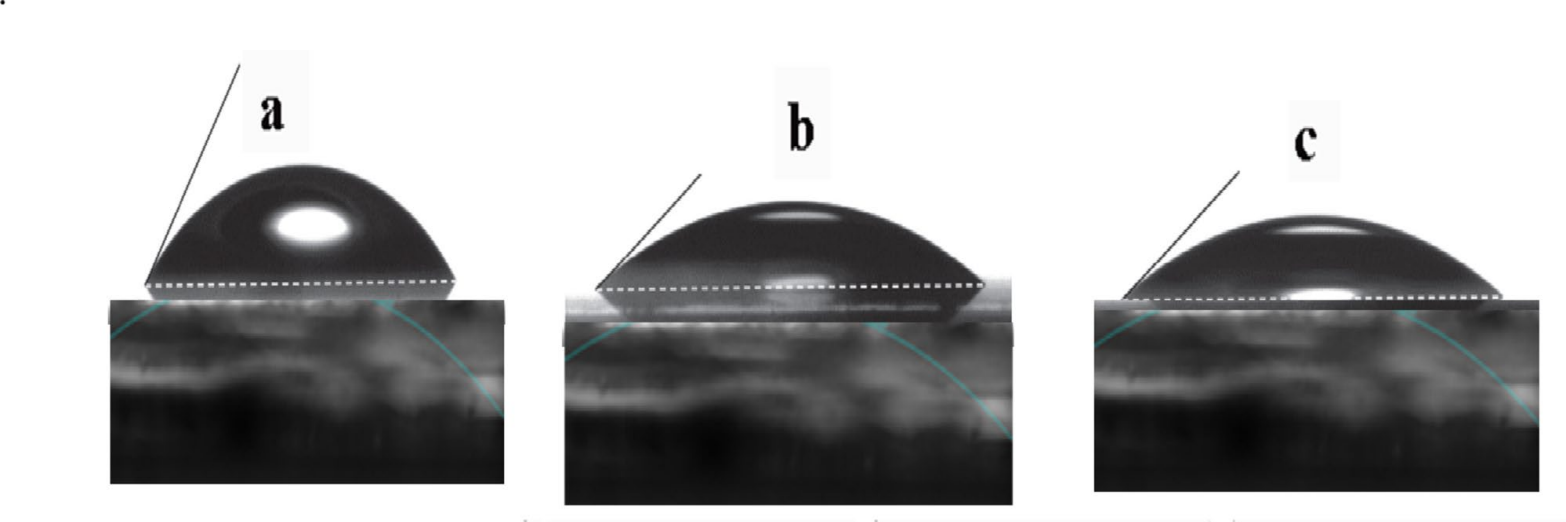
Relevant pictures of contact angles were extracted from the movie clips emulating interaction between (**a**) VR13, (**b**) β-CD/VR13 complex by encapsulation method and (**c**) β-CD/VR13 complex by kneading method droplet and viscose fabrics.

 The contact angle is a measurement of the likelihood that the surface will get wetted by water. It is the angle at the interface where water, air, and solid meet. High contact-angle values indicate the surface’s hydrophobicity to repel water, while low contact-angle values indicate the tendency of the water to be absorbed on the surface. Figure 5 displayed the contact angle representing the interaction of droplets of VR13, β-CD/VR13 complex by encapsulation method and β-CD/VR13 complex by kneading method and viscose fabric. Results show that the original vat exhibits a contact angle of 84° on viscose fabric. On the other hand, β-CD/VR13 complex prepared by either encapsulation or kneading method, demonstrates a strong tendency for viscose and appears to have a lower contact angle (approximately 40° and 34° respectively). The visual analysis also revealed that the droplet of β-CD/VR13 complex is absorbed by viscose fabric remarkably faster than VR13, and because of this fact, the contact angle is dynamic simulation value. The strong affinity of β-CD/VR13 for viscose may be attributed to the reduction of free surface energy of viscose and β-CD/VR13.

The hydrophilic outer surface of β-CD enhances the wettability of VR13, as demonstrated by contact angle measurements. The contact angle reduced from 84° for pure VR13 to roughly 34° for the kneading approach and 40° for the encapsulation method, showing a better interaction between the dye and water. Generally speaking, it was assumed that the hydrophilic outer surface of β-CD molecule make it is easier for VR13 to be included in the cavity as a result, after an inclusion complex forms, VR13’s aqueous solubility and wettability are significantly increased. It seems that original vat dye under the condition of analysis without the conventional preparation process (reduction and oxidation) could not accomplish the reasonable dye dissolution to be adsorbed by viscose fabrics.

### Morphology and size distribution

**Fig. 6:**
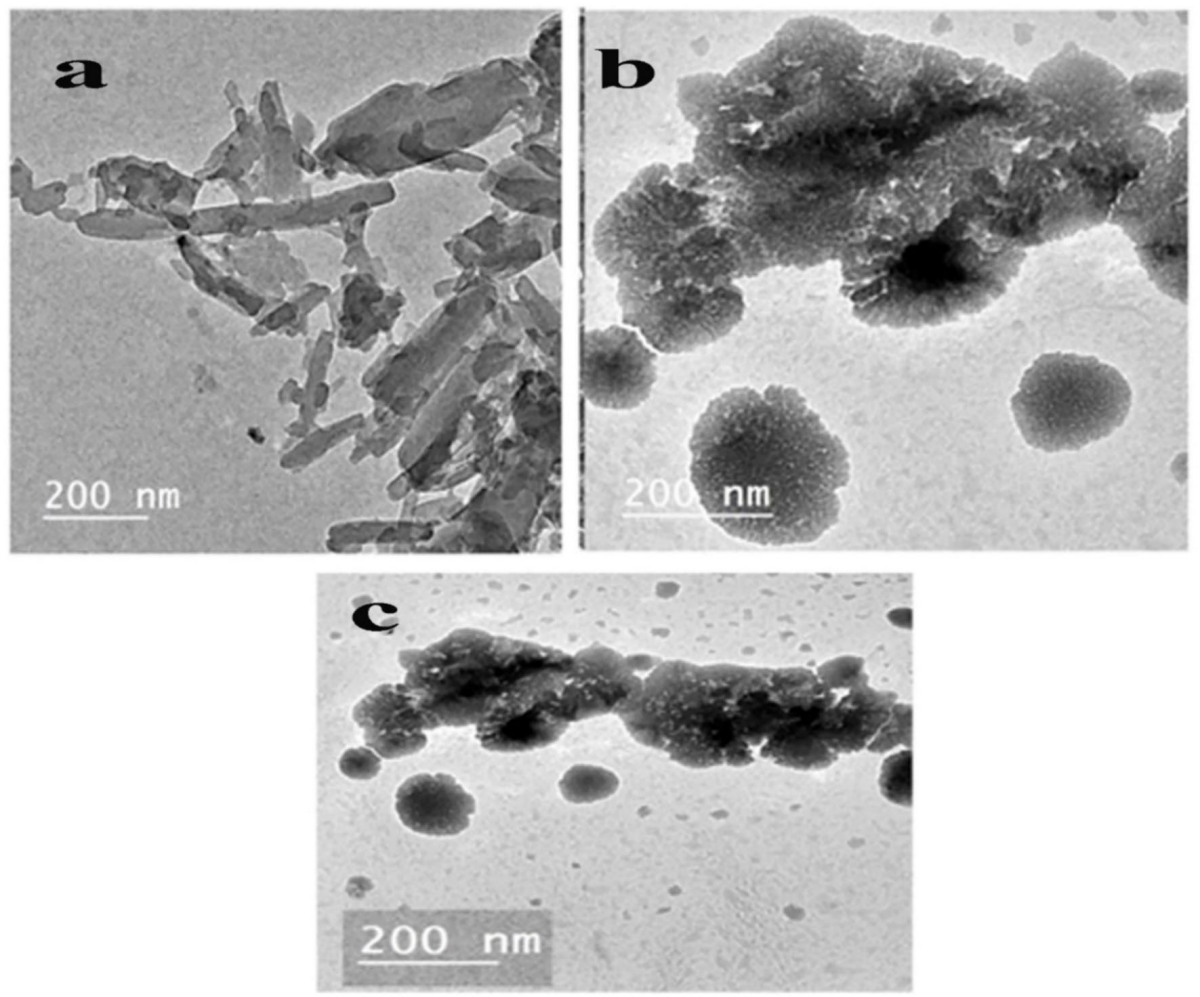
TEM morphological observation of (**a**) VR13, (**b**) β-CD/VR13 complex by encapsulation method and (**c**) β-CD/VR13 complex by kneading method.

TEM Morphological observation of VR13, β-CD/VR13 complex by encapsulation method, and β-CD/VR13 complex by the kneading method has been shown in Fig. 6.TEM clearly shows the difference of surface morphology of original VR13 and β-CD/VR13 inclusion complexes. It is evident that the original VR13 consisted of rod-shaped, elongated, agglomerated particles that are more difficult to disperse and are firmly bonded together. On the other hand, Fig. 6 (b, c) shows the inclusion of rod-like particles of VR13 into the honeycomb porous structure cavity of β-CD. One piece of evidence that supports the inclusion of vat dye moiety into the β-CD cavity is the modification of the structural characteristics of inclusion complexes from β-CD/VR13 and VR13.

The TEM micrographs were intended to visualize the morphological changes in the inclusion complex. The honeycomb structure we observed is consistent with our XRD findings on β-cyclodextrin inclusion complexes, where such formations can result from the disruption of the crystalline structure, generating more amorphous regions. These amorphous regions, in turn, may contribute to the enhanced dissolution behavior observed in our study^24^. The honeycomb-like structure might also be explained by the interaction of β-cyclodextrin with Vat Red 13 during the encapsulation process. This interaction could lead to a porous network formation due to the partial solubilization of the dye and its subsequent redistribution within the complex. Such a structure might increase the surface area available for solvent interaction, contributing to the observed improvement in solubility^24^.

**Fig. 7:**
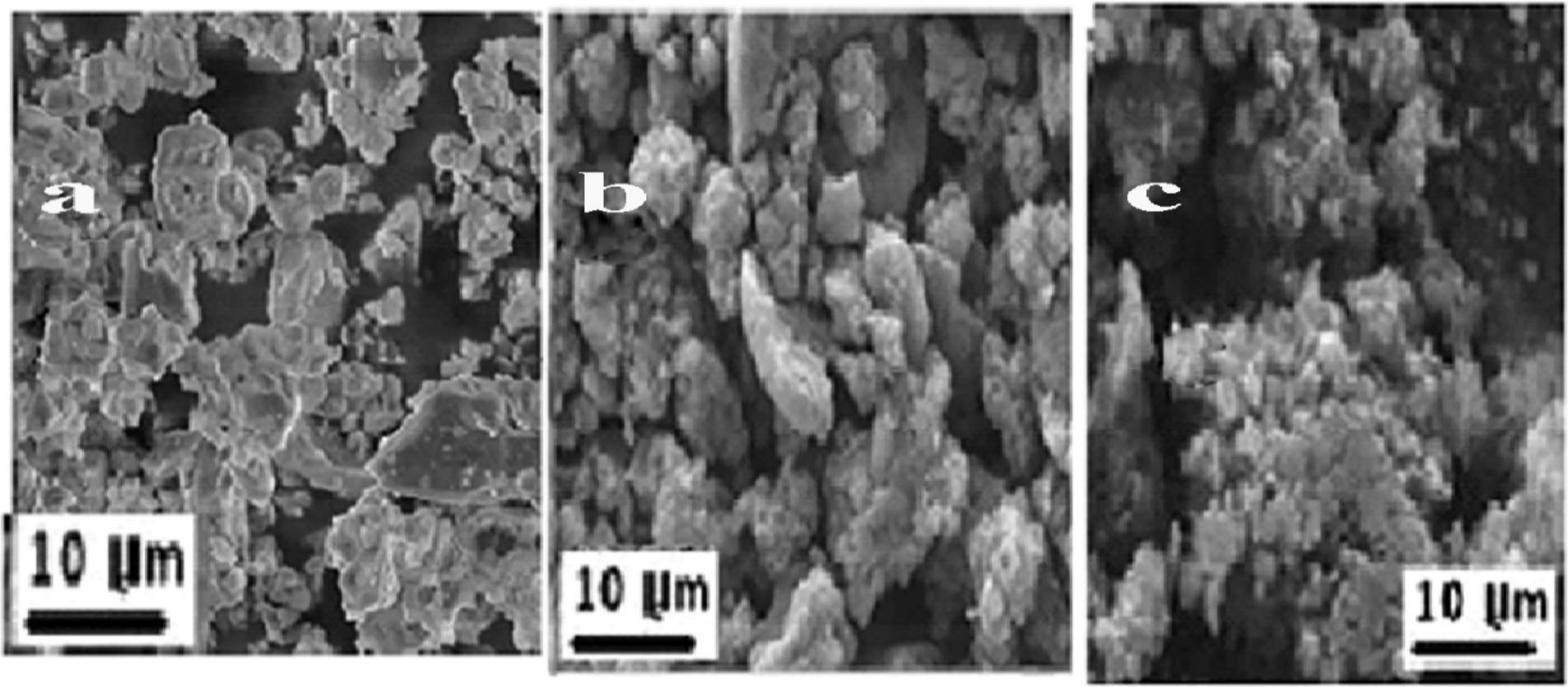
SEM morphological observation of (**a**) VR13, (**b**) β-CD/VR13 complex by kneading method and(**c**) β-CD/VR13 complex by encapsulation method.

The structural distinctions between pure Vat Red 13 (VR13) and its β-cyclodextrin (β-CD) inclusion complexes are clearly seen in Fig. 7’s SEM findings. High crystallinity and poor solubility are shown by the rod-like, aggregated particles seen in pure VR13 (Fig. 7a). This is consistent with the high crystallinity index found in XRD data. The kneading method of the β-CD/VR13 complex (Fig. 7b) shows a porous structure with less particle aggregation, indicating that mechanical grinding partially disrupted the crystalline structure. Better dispersion and encapsulation within the β-CD cavity are indicated by the smoother texture and smaller, more evenly dispersed particles of the β-CD/VR13 complex obtained using the encapsulation approach (Fig. 7c). These morphological changes enhance solubility and dissolution by reducing particle size and crystallinity, increasing the surface area for solvent interaction. Both techniques successfully lower particle size and agglomeration in comparison, however the encapsulating method creates a more porous structure while the kneading method produces a slightly smoother surface. The different processes of interaction during preparation are responsible for these variations. The dyeing performance is further enhanced by the more homogenous complex produced by the encapsulation process. SEM results support XRD and contact angle data, showing how β-CD alters VR13 morphology to improve solubility and textile application effectiveness.

**Fig. 8:**
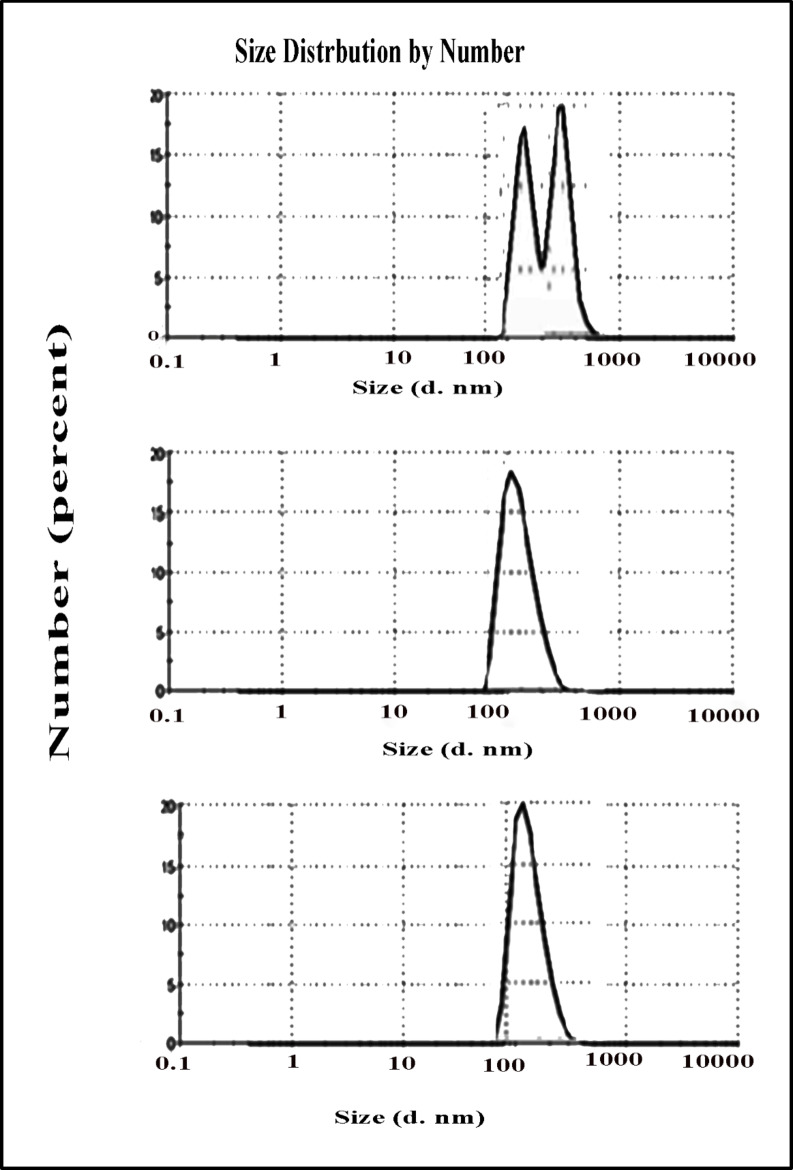
particles size distribution of (**a**) VR13, (**b**) β-CD/VR13 complex by encapsulation method and (**c**) β-CD/VR13 complex by kneading method.

It is obvious from Fig. 8 that PSD of original VR13 before inclusion into β-CD is bi-modal with a substantial fraction in the size range (> 500 nm) and a minor fraction in the size range (< 500 nm). It is important to note that, in contrast to the β-CD/VR13 complex (0.2–0.3), the high PDI value of ~ 0.5 of the original VR13 showed a strong agglomeration and a broad particle size (data not shown). This may be due to the aggregate of poorly water soluble VR13 particles as well as the vat–vat hydrophobic interaction. Analysis of particle size distribution confirms improved solubility. The average particle size of VR13 dropped significantly from > 500 nm (pure VR13) to < 300 nm after kneading and encapsulation. The polydispersity index (PDI) improved dramatically, showing better dispersibility and less aggregation, which directly contributes to increased solubility.

Based on the findings, it was shown that the encapsulation of VR13 into β-CD cavity causes an enormous reduction in the particle size with a single modal distribution of particles (Fig. 8b, c), as well as PDI which in turn, enhances the dispersibility and dissolution of vat particles. This may be explained by the intensified charges around vat surface provided by host–guest inclusion complex of β-CD due to its hydrophobic central cavity and hydrophilic outer surface in structure. Figure 8 shows that the β-CD/VR13 complex had significantly lower particle size and polydispersity index (PDI) compared to pure VR13. The amorphous structure produces smaller, more homogeneous particles, which improves dispersion and dissolution efficiency.

#### XRD

**Fig. 9:**
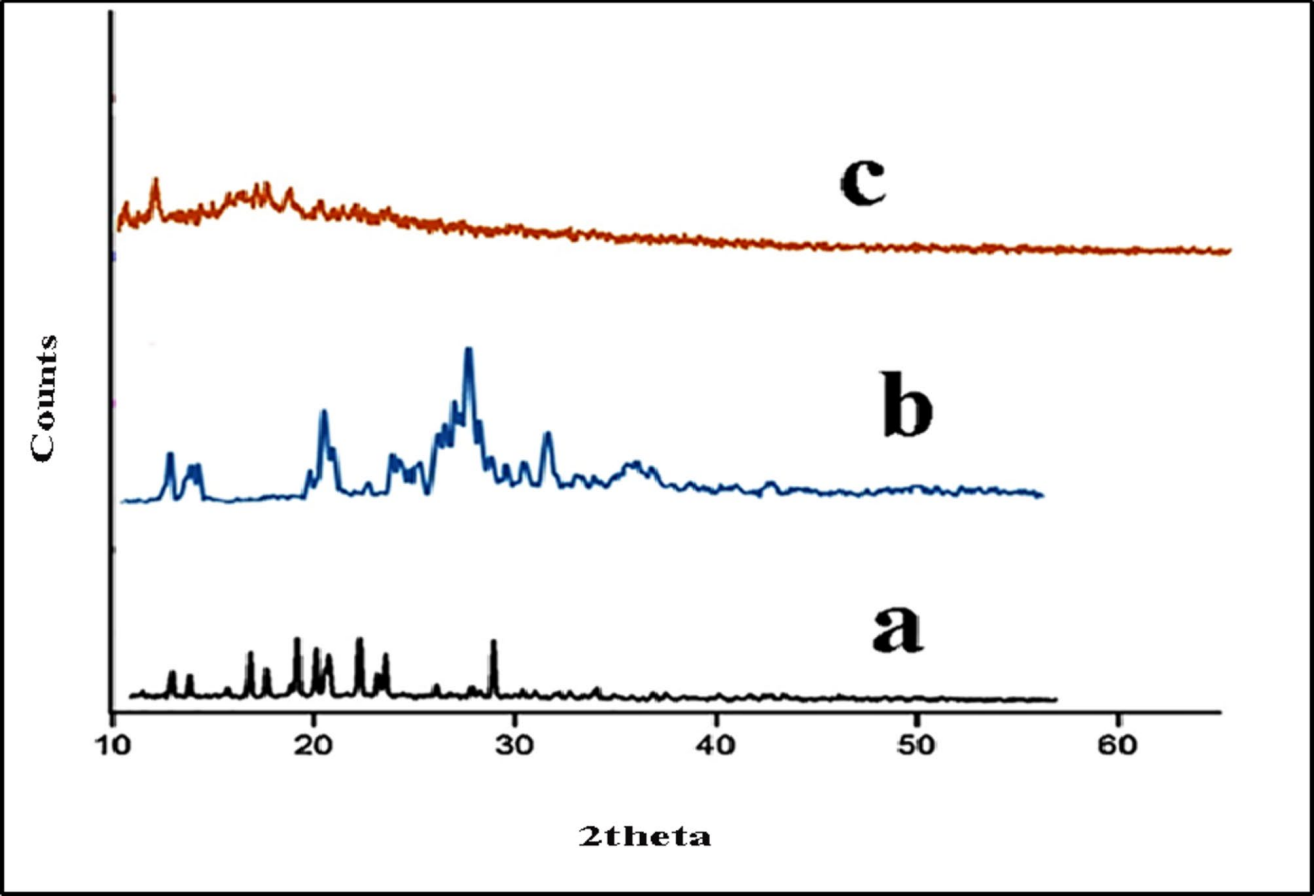
X-ray diffraction pattern of (**a**) β-CD, (**b**) VR13 and (**c**) β-CD/VR13 complex by kneading method.

The crystalline nature of the materials was ascertained and confirmed using the X-ray diffraction technique. The X-ray diffraction patterns of VR13, β-CD and β-CD/VR13 are shown in Fig. 9. From the distinctive peaks that show up at a different diffraction angle in the XRD pattern of pure VR13 (Fig. 9b), it can be deduced that the original vat color is still preserved in its crystalline form.

Furthermore, the XRD pattern of β-CD (Fig. 9a) showed a significant peak at 2θ = 12.66°, 15.96°, 16.12°, 18.24°, 19.00°, 21.14°, 23.00°, 24.22°, 25.7°, and 27.10°, which may indicate that they are crystalline in nature. Conversely, the XRD pattern β-CD/VR13, which was created by the kneading approach (Fig. 9c), is entirely amorphous, which indicates that VR13 has been effectively included into the β-CD molecule. Thus, VR13 is effectively included with β-CD. Beyond a rise in solubility, the crystalline character of the VR13 molecules is reduced during kneading processes to produce a highly energetic amorphous state, which could be another factor contributing to the accelerated dissolution of the inclusion complex^41,42^. The XRD results support the findings from Table 1; Fig. 1, confirming that the solubility of VR13 rose linearly with β-CD concentration up to 0.04 M, indicating the formation of amorphous areas (Fig. 1; Table 1). At larger concentrations of β-CD, water-insoluble complexes may form, leading to a decrease in solubility. These findings show that amorphous areas in the β-CD/VR13 inclusion complex improve dissolution rate by increasing surface area, boosting molecular mobility, and facilitating better dispersion.

In light of this, the samples’ crystallinity index (CI) was calculated in accordance with the Segal method^34^. VR13, β-CD, and β-CD/VR13 were found to have CI-Segal, of 31.5%, 33%, and 19%, respectively. The X-ray diffraction (XRD) study showed a considerable drop in the crystallinity index (CI) of VR13 when combined with β-CD, from 31.5% for pure VR13 to 19% for the β-CD/VR13 complex (Table 2). This decline suggests the emergence of more amorphous regions, disrupting VR 13’s regular crystalline structure.

Amorphous regions have larger surface energy and irregular molecular configurations than crystalline structures, leading to increased surface area for solvent interactions. This increases the accessible surface area for solvent interaction, resulting in quicker wetting and dissolution.

XRD investigations show that VR13’s crystallinity index (CI) decreases significantly when complexed with β-CD, from 31.5% for pure VR 13 to 19% for the inclusion complex. This transition results in more amorphous areas, which promotes better solvent interaction and faster dissolution.

The degree of crystallinity (Xc) followed the same trend of Segal method as was shown in Table 2. Xc and D of VR13 have been decreased drastically after inclusion into β-CD which are consistent with data of XRD patterns. This observation in our study indicated that β-CD is more influential towards the crystalline regions of VR13.

**Table 2:** Effect of β-CD on crystallite size (D), interplanar distance/d-spacing (d), full width at half Maximum(FWHM), degree of crystallinity(Xc) by lorentzian function, crystallinity index the Segal method(CI Segal), crystallinity index (IR-CI), total crystallinity index (TCI) and hydrogen bond intensity (HBI) of β-CD/VR13 inclusion complex.

Sample	XRD						FTIR		
	2${\rm \theta}\:\circ$	D(A$\circ$ )	d- spacing (Å)	FWHM	Xc	CI-Segal	CI-IR	TCI	HBI
**VR13**	15.9 °	26.01	4.74223	0.1	44%	31.5%	0.55	0.39	0.19
	21.14°	27.33	4.770495	0.1					
	27.10 °	27.71	3.5432	0.1					
**β-CD**	15.9 °	29.68	4.80233	0.1	47%	33%	0.74	0.71	1.07
	21.14°	28.53	4.24532	0.1					
	27.10 °	27.19	3.66977	0.5					
**β-CD/VR13**	15.9 °	18.18	4.85222	0.1	24%	19%	0.044	0.036	1.11
	21.14°	17.37	4.28444	0.1					
	27.10 °	16.23	3.93456	0.3					

#### ^1^**H nuclear magnetic resonance analysis**

^1^H NMR revealed the formation of inclusion complexes between Vat Red 13 (VR13) and β-cyclodextrin (β-CFig. ure 10 shows NMR spectra for pure VR13 (a), β-CD/VR13 prepared via encapsulation (b), and β-CD/VR13 prepared via kneading (c). In the spectrum of pure VRFig. ig. 10a), characteristic signals corresponding to the aromatic protons of the anthraquinone core structure, such as aromatic C-H and quinone groups, are observed in the range of δ 7.5–8.5 ppm, along with characteristic peaks for its carbonyl (C = O) and C-N functional groups. These peaks depict VR13’s natural chemical environment in its free, non-complexed condition. The spectrum of pure β-CD (not shown but inferred from literature) has resonances for H-3 (≈ 3.8 ppm) and H-5 (≈ 3.5 ppm) protons inside the hydrophobic cavity, as well as H-1, H-2, H-4, and H-6 protons on the hydrophilic outside^43^. Forming inclusion complexes with β-CD results in considerable changes and widening of major proton signals in both kneading and encapsulation samplFig. gs. 10b and 10c). The^1^H NMR spectra of β-CD/VR13 generated using encapsulation techniqFig. ig. 10b) differs significantly from that of pure VR13. The signals for the H-3 and H-5 protons of β-CD (placed inside the cavity) show significant up field shifts (e.g., H-3 shifts from ≈ 3.8 ppm to ≈ 3.6–3.7 ppm), showing that the aromatic moieties of VR13 are imprisoned within the β-CD cavity. The most significant shift is detected for H-5 (Δδ = 0.3 ppm), which is placed deeper in the cavity, supporting the notion of host-guest interaction. VR13’s hydrophobic groups’ closeness to β-CD protons causes shielding or de-shielding effects, resulting in this disturbance. VR13’s aromatic protons (6.5–8.5 ppm) show lower intensity in complex spectra, indicating restricted mobility and partial inclusion within the β-CD cavity. Similarly, the spectra of β-CD/VR13 produced by the kneading approaFig. ig. 10c) shows analogous chemical shift modifications for β-CD’s internal protons. However, the amplitude of these shifts appears to be slightly different from the encapsulation strategy, which could indicate variances in the inclusion efficiency or geometric layout of the host-guest complex. Moreover, the external protons of β-CD (H-1, H-2, and H-4) show minor modifications in their chemical shifts in both approaches of inclusion complexes. This is consistent with their placement on the exterior of the cyclodextrin cavity, having limited contact with the guest molecule. These NMR observations support the successful creation of inclusion complexes between VR13 and β-CD using both preparation methods. The anthraquinone core of VR13 is accommodated within the hydrophobic cavity of β-CD, as illustrated in scheme 2. This is confirmed by the selective chemical shift changes of the internal cavity protons of β-CD, as well as modifications in the guest molecule signals. The comparative analysis of spectra from both preparation methods indicates that, while both techniques successfully produce inclusion complexes, subtle differences in spectral features may reflect variations in inclusion efficiency or the precise geometric arrangement of the guest molecule within the host cavity, which could potentially explain the differences in dissolution enhancement observed in other characterization studies^44,45^.

**Fig. 10:**
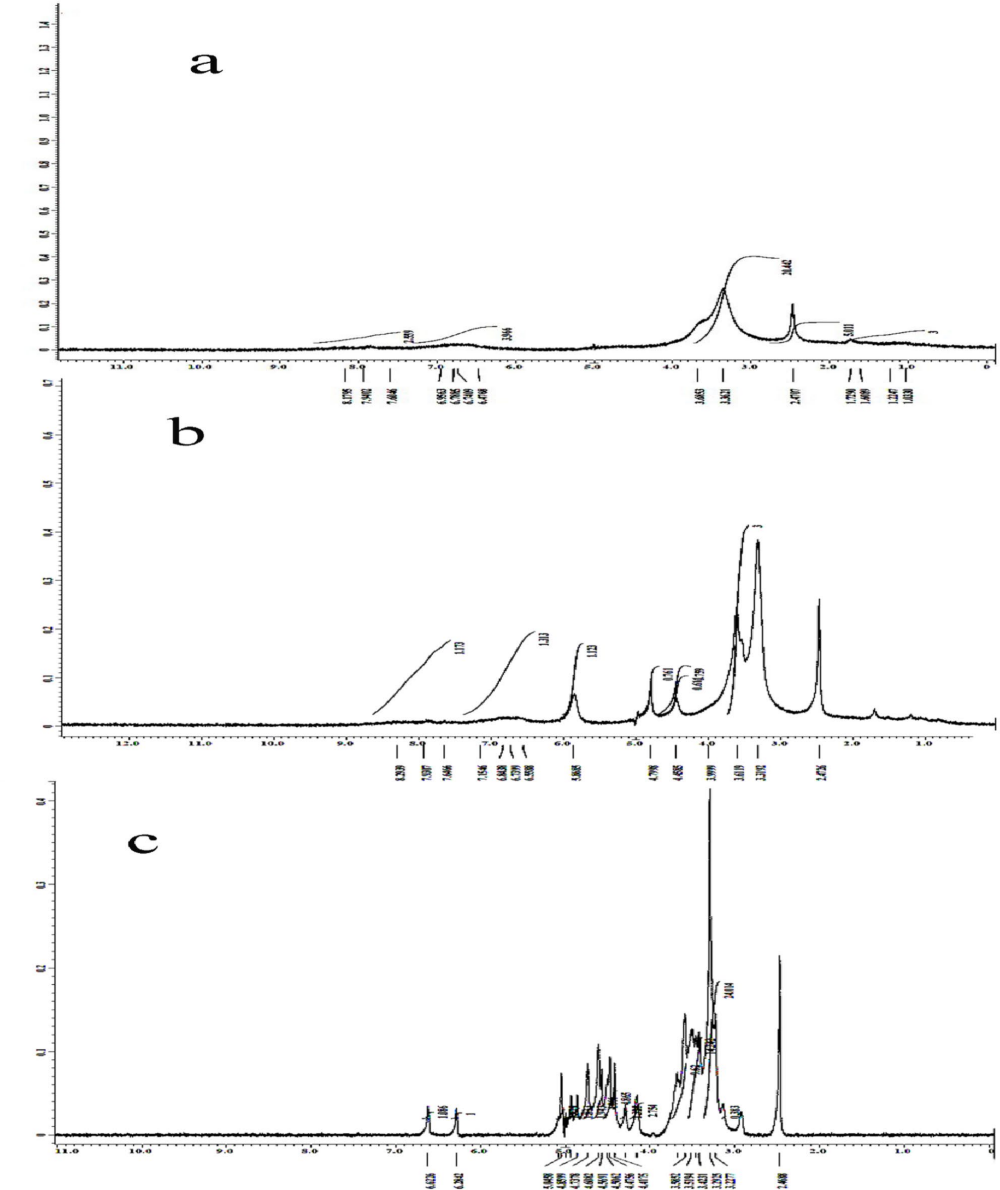
^1^H NMR spectra of **a**) VR13, **b**) β-CD/VR 13 encapsulation method, and **c**) β-CD/VR13 kneading method.

## Dyeing performance

### **Effect of varying the concentration of reducing agent on the colorimetric values of β-CD/VR13 dyed viscose fabric**

The goal of this endeavour is to eliminate or reduce the hazardous reducing agent account that affects both humans and the environment. By reducing its use by 75%, the creation of these harmful byproducts is greatly decreased, resulting in lower chemical oxygen demand (COD) and less water contamination from dye effluents^18^. Beyond environmental benefits, this approach also offers significant economic advantages. Since sodium dithionite is one of the most expensive components in vat dyeing, its reduction results in considerable cost savings for textile manufacturers. Additionally, the faster dissolution of the β-CD/VR13 inclusion complex shortens processing times and lowers energy consumption, further enhancing the overall economic viability of the process^9,10,16^ .

One popular technique to overcome the severe conventional conditions of vat coloration and significantly improve the dissolving of vat dyes is to modify VR13 by inclusion into the β-CD cavity. With this in mind, different concentrations of sodium hydrosulphite, a reducing agent, have been applied to viscose fabric in vat coloration processes, ranging from 25 to 100% depending on the weight of the corresponding value for VR13 and β-CD/VR13. It is important to note that 20 g/l (100%) is the recommended reducing agent concentration for traditional vat dyeing. Table 2appears to indicate that the unfavorable reducing agent conditions (25–75%), which result from a decreased sodium hydrosulfite quantity, are insufficient to complete and reach the vat fixation balance necessary to produce dyed samples with traditional VR13 that can bring about reasonable color strength^10^. For example, the decrease of the appropriate reducing agent is accompanied by sudden decrease of color strength from 21.51 to 6.82, from 21.51 to 5.36 and from 21.51 to 3.95 for 75%, 50%, and 25% of appropriate reducing agent, respectively. It can be inferred that the standard VR13 appears to require the standard quantity of reducing agent and cannot be processed in unfavorable conditions. Conversely, β-CD/VR13 inclusion complex, either by kneading method or encapsulation accelerate the vat fixation rate with fabric dyeing uniformity and brilliancy. The results showed that the dyeing process with the β-CD/VR13 inclusion complex is unaffected by the decrease in sodium hydrosulfite. Generally speaking, the β-CD/VR13 inclusion complex’s lowered sodium hydrosulfite content of 25% is sufficient to accomplish and reach the dyeing fixation balance that can result in reasonable color performance, which has been determined to be industrially acceptable.

For instance, the decrease of the appropriate reducing agent is accompanied by marginal decrease of color strength from 29.64 to 27.85, from 29.64 to 26.71 and from 29.64 to 26.6, in case of kneading method, and from 27.57 to 26.45, from 27.57 to 25.69 and from 27.57 to 25.05, in case of encapsulation for 75%, 50%, and 25% of appropriate reducing agent, respectively. Furthermore, the color strength of the dyed samples containing β-CD/VR13 inclusion complex with 25% sodium hydrosulfite concentration was found to be higher than that of the traditional VR13 with 100% of the reducing agent. Vat dyes are oxidized dispersions that require pre-reduction to ensure solubility and affinity for cellulose. The solution is transferred to impregnation boxes using this reductive process, known as vatting, usually with the use of indigo. The type of dye and the pick-up of the reductive solution determine the reducing agent and alkali to be employed. The water-insoluble dyes containing carbonyl groups can be converted into a soluble form by reduction in an alkaline condition. The resulting ‘leuco compound’ is then absorbed by cellulose and undergoes subsequent oxidation, which returns the original form of the insoluble vat dye to the fiber. In addition to poor exhaustion and very little diffusion inside the fiber, the unfavorable conditions caused by employing lower amounts of sodium hydrosulfite with standard VR13 also result in larger deposits of insoluble dyes on fabrics. In general, decreased sodium hydrosulfite concentrations are unable to facilitate dye penetration into cellulosic fibers. This phenomenon may be caused by diminishing the stability of alkaline reducing agent solutions. This may have caused an excessive number of loose dyes to be washed out. On the other hand, when VR13 is physically mixed with β-CD, the solubility increases, the crystalline nature of VR13 molecules decreases to form highly energetic amorphous, and the local solubilization impact of the carrier molecules, all contribute to the improved dissolution. This may be substantially offsetting the decrease in sodium hydrosulfite, which forces VR13 to be reduced to the proper concentration in order to have adequate fiber affinity and solubility. The colorimetric results for dyed viscose samples using conventional VR13, β-CD/VR13 complex by encapsulation method, and β-CD/VR13 complex by kneading method at varying sodium hydrosulfite concentrations ranging from 25 to 100% are displayed in Table 3. Generally speaking, it is evident that lowering the sodium hydrosulfite concentration raised the lightness (L*) of the VR13-dyed samples, indicating somewhat deeper hues at 100% concentration. Furthermore, a little, non-significant decrease in red shade was seen with decreasing sodium hydrosulfite for dyed samples containing β-CD/VR13 complex, whether by kneading or encapsulation method. Additionally, the color did not change appreciably at any of the sodium hydrosulfite doses; but, at 25% of sodium hydrosulfite, the dyed samples containing the β-CD/VR13 complex were duller and more red-shaded than the conventional ones at 100%.

**Table 3:** Effect of varying the concentration of sodium hydrosulfite on the colorimetric values of dyed viscose samples.

Sample	Reducing agent	λ	k/S	L	a	b	ΔE
**VR13**	100%	520	21.51	33.56	49.58	10.94	70.56
	75%		6.82	53.23	39.80	−6.77	56.76
	50%		5.36	57.04	37.10	−7.47	54.17
	25%		3.95	58.70	34.21	9.74	48.91
**β-CD/VR13** **(kneading)**	100%	520	29.64	23.61	56.84	13.74	81.30
	75%		27.85	27.90	54.83	11.95	79.26
	50%		26.71	28.17	53.79	12.35	77.72
	25%		26.60	29.16	51.85	10.43	74.38
**β-CD/VR13** **(encapsulation)**	100%	520	27.57	26.78	54.88	12.09	79.31
	75%		26.45	28.28	52.72	11.47	76.20
	50%		25.69	33.79	51.39	10.21	74.79
	25%		25.05	38.27	50.56	9.87	72.82

#### Fastness properties

**Table 4:** Fastness properties of fabrics dyed by VR13 and β-CD/VR13 inclusion complex.

Sample	Reducing agent	Washing fastness			Rubbing fastness		Light fastness
		Alt.	Sc.	Sw.	Wet	dry	
**VR13**	100%	4	4	4	3	3	6–7
	75%	3–4	3–4	3	3	3	6–7
	50%	3–4	3	3	2–3	3	6
	25%	3	3	3	2–3	2–3	5–6
**β-CD/VR13** **(kneading)**	100%	4–5	4–5	4–5	4	4	7
	75%	4–5	4–5	4–5	3–4	4	7
	50%	4	4	4	3–4	3–4	6–7
	25%	4	4	4	3–4	3–4	6–7
**β-CD/VR13** **(encapsulation)**	100%	4–5	4–5	4–5	4	4	7
	75%	4–5	4–5	4–5	3–4	4	7
	50%	4	4	4	3–4	3–4	6–7
	25%	4	4	4	3–4	3–4	6–7

Table 4 displays the dyed viscose samples’ light, rubbing, and washing fastness at varying sodium hydrosulfite concentrations with the conventional VR13 and β-CD/VR13 inclusion complex. It is evident that as compared to the traditional VR13, the inclusion complex-colored samples displayed superior fastness properties. It has also been shown that reducing the suitable concentration of sodium hydrosulfite has led to a minor loss in the fastness qualities of the traditionally dyed samples. This might be the result of more loose dyes being deposited on the surface of viscose fabrics, which are impacted by a drop in sodium hydrosulfite dosage and insufficient solubility and fiber affinity. Overall, the colored samples from the inclusion complex exhibited good light and washing fastness, with ranges of 6–7 for light fastness and 4–5 for washing fastness. Nonetheless, it was discovered that the color fastness, which varied from 3 to 5, was industrially acceptable for both wet and dry rubbing. Furthermore, the fastness properties of dyed samples with β-CD/VR13 inclusion complex are not significantly affected by a decline in sodium hydrosulfite dose. Based on the above results few points May be concluded; (a) by encapsulating conventional VR13 into β-CD, the vat dyeing process can be accelerated under unfavorable conditions by using less hazardous sodium hydrosulfite; (b) using conventional amounts of sodium hydrosulfite is necessary to achieve the proper dye solubility and fiber affinity that can result in a reasonable color strength; (c) β-CD has a greater influence on the vat dyeing process under unfavorable conditions. The amount of dissolved VR13 and its rate of dissolution both rise quickly as a result of the inclusion complex. The VR13 molecules undergo a reduction in crystalline nature during the kneading process, resulting in the production of an extremely energetic amorphous form. Furthermore, the hydrophilic exterior surface of β-CD makes VR13 molecules more wettable when included; and d) dyed samples containing β-CD/VR13 inclusion complex with 25% sodium hydrosulfite concentration were found to have better color performance than traditional VR13 with 100% of the reducing agent. Our investigation further revealed that combining vat red 13 with β-cyclodextrin improves its solubility and dissolving rate. This enhancement reduces the quantity of reducing agent needed by roughly 75%, reducing environmental impact and production costs while retaining outstanding dyeing performance. The β-CD/VR13 inclusion complex outperforms standard reducing agents at 25% concentration, making it industrially feasible. This ensures that the color strength, lightfastness, and washing fastness qualities are consistent with or better than those obtained using standard procedures. The durability of this approach makes it ideal for large-scale industrial use without sacrificing quality. 

## Conclusion

Using kneading and encapsulating procedures, the β-CD/VR13 inclusion complex was effectively created. The effective development of the inclusion complex was confirmed by extensive characterization with FTIR, UV-Vis spectra, contact angle measurements, TEM, XRD, and particle size distribution studies. UV-Vis absorption results showed a considerable increase in absorbance for β-CD/VR13 compared to conventional VR13, confirming the inclusion complex’s improved solubility in liquid. Phase solubility experiments confirmed that β-CD improves VR13 solubility. VR13 solubility rose linearly with β-CD concentration up to 0.04 M, indicating a Bs-type system as stated by Higuchi and Connors. Higher β-CD concentrations led to the development of water-insoluble complexes, resulting in lower solubility. The kneading and encapsulation procedures yielded apparent stability constants (K1:1) of 147.9 mol⁻¹ and 129.4 mol⁻¹, respectively, indicating that the 1:1 stoichiometric ratio between β-CD and VR13 is stable. The inclusion complex dramatically reduced the quantity of reducing agents required for vat dyeing viscose fabric, resulting in equal or improved color performance with only 25% of the normal reducing agent concentration as compared to conventional VR13. This reduction not only reduces the environmental impact of vat dyeing methods, but it also increases economic viability by cutting manufacturing costs. The β-CD/VR13 inclusion complex has outstanding fastness qualities, such as light, rubbing, and washing, making it suitable for industrial applications. The β-CD/VR13 inclusion complex can improve the dissolving rate and solubility of vat dyes, reduce the use of hazardous reducing agents, and enhance the sustainability of textile dyeing processes. Future study should optimize preparation processes and explore the potential of β-CD derivatives to improve performance and expand applications.

## Data Availability

All data generated or analyzed during this study are included in this manuscript.
